# Discovery and Characterization of Mannan-Specialized GH5 Endo-1,4-β-mannanases: a Strategy for Açaí (*Euterpe oleracea* Mart.) Seeds Upgrading

**DOI:** 10.1021/acs.jafc.4c07018

**Published:** 2024-12-16

**Authors:** Roberta P. Espinheira, Kristian Barrett, Lene Lange, Ayla Sant’Ana da Silva, Anne S. Meyer

**Affiliations:** † Divisão de Catálise, Biocatálise e Processos Químicos, Instituto Nacional de Tecnologia, Av. Venezuela 82, Rio de Janeiro 20081-312 ,Brazil; ‡ Programa de Pós-graduação em Bioquímica, Universidade Federal do Rio de Janeiro, Av. Athos da Silveira Ramos 149, Rio de Janeiro 21941-909 ,Brazil; § Department of Biotechnology and Biomedicine, Technical University of Denmark, So̷ltofts Plads 221, 2800 Kgs Lyngby, Denmark; ∥ LL BioEconomy, Research & Advisory, Karensgade 5, 2500 Copenhagen, Denmark

**Keywords:** açaí seeds, linear β-mannan, endo-1,4-β-mannanase, enzyme discovery, enzyme characterization.

## Abstract

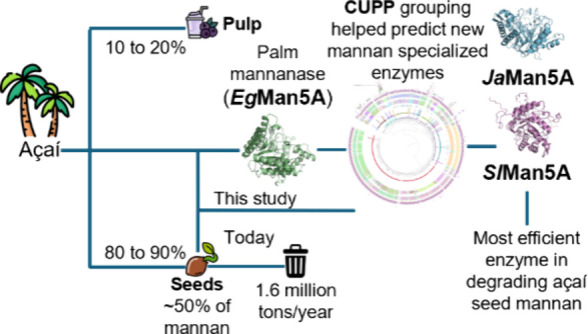

The pulp of açaí palm fruits (*Euterpe oleracea* Mart.) is a valuable export commodity in Brazil. Its production generates 1.6 million tons/year of açaí seeds, a resource largely wasted. The seeds consist mainly of linear β-mannan, offering potential for prebiotic β-mannan-derived oligomers and mannose production. However, the crystalline structures of β-mannan hinder enzymatic hydrolysis. This study aimed to discover and characterize fungal enzymes targeting açaí seed β-mannan using a palm β-mannanase (*Eg*Man5A) as a guide. Recombinant expression, enzyme optimization, kinetics, substrate specificity, and structural modeling were performed. The two fungal enzymes, *Ja*Man5A and *Sl*Man5A, were found to be specific for unsubstituted mannan, showing no activity toward galacto- and glucomannan. Among them, *Sl*Man5A showed the highest activity on açaí seed β-mannan (∼24 U/mg) and other unsubstituted mannan substrates, likely due to its greater thermal robustness. These results provide valuable insights into β-mannan specificity contributing to the sustainable valorization of açaí seeds.

## 1 Introduction

The açaí palm (*Euterpe oleracea* Mart.) produces one of the most valuable fruits of the Brazilian Amazon, known as açaí fruits.^1^ These dark purple fruits are processed to extract their pulp, which is either consumed locally or subjected to drying or freezing processes for export and use in various foods, such as smoothies, yogurt, and confectionery. In 2022, Brazil produced 1.9 million tons of açaí, representing a 60% increase when compared to 2015.^2^ Unfortunately, açaí production negatively impacts the environment, as the edible part represents only 15% of the fruit,^3,4^ leaving about 1.6 million tons of seeds as waste per year, which is largely disposed of in landfills.^5^ Although currently discarded, açaí seeds contain about 50% of β-mannan,^3^ a high amount also when compared with other common β-mannan-containing agro-industrial residues.^6,7^ This feature makes the açaí seed a valuable natural source of β-mannan and β-mannan oligosaccharides, which are known to exert prebiotic activity.^8^

β-mannans are classified as galactomannans, glucomannans, galactoglucomannans, and linear mannans.^9^ As the carbohydrate composition of açaí seeds has shown a low percentage of galactose and glucose, and its crystalline profile is compatible with type 1 linear β-mannan,^3^ the β-mannan of those seeds is inferred to be organized in the linear form creating crystalline assemblies,^10^ like the type of β-mannan found in ivory nut.^9^

The linear β-mannan of the açaí seeds could therefore be used to directly produce β-mannooligosaccharides (β-MOS) and mannose by enzyme catalyzed depolymerization. However, the packed β-mannan chains confer crystallinity and recalcitrance to enzymatic attack,^11^ impairing this task.

Endo-1,4-β-mannanases (E.C. 3.2.1.78) catalyze the cleavage of the internal bonds of 1,4-β-D-mannans.^12^ According to the Carbohydrate-Active enZymes (CAZy) database (http://www.cazy.org/), endo-1,4-β-mannanases are grouped into four families of glycosyl hydrolases (GH), GH5, GH26, GH45, and GH113, with GH5 and GH26 families harboring the majority of endoacting β-mannanases.^13^ Different factors influence the specificity of endo-1,4-β-mannanases toward β-mannans. For example, studies have shown that the number and specificity of binding subsites, along with the catalytic mechanism, affect hydrolytic efficiency and specificity, as seen in comparisons of GH5 and GH26 endo-1,4-β-mannanases.^12,14−16^

Few studies have examined the enzymatic hydrolysis of β-mannan in untreated açaí seeds, and those that have reported low yields after 24–72 h of hydrolysis.^3,17^ These low yields of açaí seed enzymatic hydrolysis are associated not only with the recalcitrant nature of the material but also with the fact that little is known about the specificity of β-mannanases toward linear mannan. Thus, discovering more specialized endo-1,4-β-mannanases and understanding the biochemical mechanisms of linear mannan hydrolysis are essential for the sustainable use of açaí seeds.

Today, various bioinformatics tools assist in enzyme discovery. Carbohydrate-active enzymes (CAZymes) are classified into families based on sequence and three-dimensional fold. Although extremely useful, this classification does not directly identify enzyme function as several CAZy families contain members that catalyze different reactions, clustering enzymes with diverse molecular functions. The family and subfamily descriptions in the CAZy database can be further refined by nonalignment tools such as the peptide-based clustering method, which identifies conserved unique peptide patterns (CUPP). The CUPP algorithm clusters protein sequences based on the division of proteins into small peptide fragments of eight amino acids in length (of which two are ambiguous) followed by comparison and grouping of these peptide fragments against a library of conserved peptide fragments that represent different CUPP groups. This approach thus enables further subclassification of enzymes into groups of functional similarity.^18,19^

Therefore, the aim of this study was to identify fungal endo-1,4-β-mannanases potentially specialized for linear mannan hydrolysis. Fungal β-mannanases were specifically selected for their broader versatility in degrading complex, recalcitrant organic substrates compared to other enzyme sources.^20^ CUPP was used as a tool for clustering fungal enzymes along with a sequence of an uncharacterized β-mannanase from the genome of *Elaeis guineensis* (oil palm)—considered to be a close botanical relative to *E. oleracea* Mart. (açaí palm) for which there is no genome sequence available. Additionally, the study aimed to clone, express, and purify the palm and the fungal enzymes and to biochemically characterize them. By doing so, this study seeks to enhance the understanding of enzyme-catalyzed hydrolysis of linear β-mannan and provide insights for developing new strategies for the sustainable utilization of açaí seeds.

## 2 Materials and Methods

### 2.1 Chemicals and Materials

Mannan (ivory nut), mannan of approximate degree of polymerization of 15—prepared by controlled hydrolysis of carob galactomannan with β-mannanase and α-galactosidase (1,4-β-D-mannan), glucomannan (konjac; low viscosity), and GH26 endo-1,4-β-mannanase (*Cellvibrio japonicus*) were purchased from Megazyme (Wicklow, Ireland). BGM “Amano” 10, a β-mannanase preparation, was kindly provided by Amano Enzyme Inc. (Nagoya, Japan). All other chemicals were obtained from Sigma-Aldrich (Steinheim, Germany).

Açaí seeds were obtained from small-scale producers of açaí pulp in Amapá state, Northern Brazil (Bailique Archipelago, Amazon River mouth–N 00°48.304’121 W 050°10.348’), and the research activity was registered in the Brazilian National System of Management of Genetic Heritage and Associated Traditional Knowledge (SisGen) under the access number #AB49D01.

### 2.2 Sequence Analysis

Fungal β-mannanases were found via a blastp (protein–protein BLAST) search (https://blast.ncbi.nlm.nih.gov/Blast.cgi) within the Nonredundant (nr) database using the sequences (XP_010911540.1, XP_008805817.3, XP_008778491.2, XP_010943133.2, XP_017700190.1) identified by Nascimento et al. of the five uncharacterized/putative endo-β-mannanase from *Elaeis guineensis* (oil palm) and *Phoenix dactylifera* (date palm) as template.^21^ Then, the top 250 resulting sequences from each alignment were analyzed with CUPP (https://cupp.info/).^18,19,22^ Sequences within the same CUPP group from palm sequences (GH5:36.1) were further examined for sequence quality and organism pathogenicity. Finally, five fungal GH5 putative endo-1,4-β-mannanase sequences were chosen for cloning based on this analysis.

### 2.3 Cloning, Expression, and Purification of Endo-1,4-β-mannanases

Cloning procedures were conducted following the methodology outlined in a previous study with some modifications.^23^ Genes encoding protein sequence (XP_010911540.1, XP_010943133.2, XP_017700190.1, XP_007324538.1, KAF8922466.1, KAF5375179.1, KDQ56244.1, and KAF8642254.1) were designed to harbor a C-terminal 6xHistidine-tag and the genes were codon-optimized for expression in *Pichia pastoris* by GenScript (Piscataway, NJ, USA), excluding their predicted signal peptides that were predicted using SignalP–6.0 (https://services.healthtech.dtu.dk/services/SignalP-6.0/). Subsequently, the genes were cloned into the pPICZαA vector (Invitrogen, Cergy-Pontoise, France), and the plasmids were linearized using FastDigest MssI (ThermoFisher, Waltham, MA, USA), and used for the transformation of *P. pastoris* X-33 via electroporation. Only the transformants with genes from *E. guineensis* (*Eg*Man5A; plant) (XP_010911540.1), *Jaapia argillacea* (*Ja*Man5A; fungus) (KDQ56244.1), and *Serpula lacrymans* (*Sl*Man5A; fungus) (XP_007324538.1) were successfully transformed.

The expression followed the protocol described previously.^24^ Culture supernatants underwent centrifugation (4000 × *g*, 5 min), followed by concentration and diafiltration against 20 mM sodium phosphate buffer, 500 mM NaCl, 20 mM imidazole, pH 7.4, using Vivaflow 200 Tangential Flow Filtration Cassettes equipped with a 10 kDa MWCO PES membrane (Sartorius, Goettingen, Germany). The concentrated supernatant was applied to a Ni^2+^ Sepharose resin (GE Healthcare, Chicago, IL, USA), pre-equilibrated with a binding buffer. Proteins were eluted with 20 mM sodium phosphate buffer, 500 mM NaCl, and 250 mM imidazole, pH 7.4. Imidazole removal utilized PD-10 columns (GE Healthcare, Chicago, IL, USA) equilibrated with 20 mM sodium phosphate buffer and 100 mM NaCl, pH 7.4.

The resulting proteins were concentrated using Vivaspin 20 Centrifugal Concentrators equipped with a 10 kDa MWCO PES membrane (Sartorius, Goettingen, Germany). The protein concentration was determined using Pierce BCA Protein Assay Kits (ThermoFisher, Waltham, MA, USA). Deglycosylation of expressed proteins involved a 3 h treatment with EndoH (New England Biolabs, Ipswich, USA) at 25 °C. Protein purity and the EndoH deglycosylation assay were assessed via SDS-PAGE gels with a 4–12% gradient and Western blotting using diluted monoclonal Anti-polyHistidine peroxidase-conjugated antibody (Sigma-Aldrich, Steinheim, Germany) and the Trans-Blot Turbo Transfer System (Bio-Rad, Copenhagen, DK). Protein aliquots were prepared by adding 10% glycerol, and the aliquots were then stored at −80 °C until use. The ProtParam tool (https://web.expasy.org/protparam/) was used to estimate the molecular weight and extinction coefficients of enzymes and N-glycosylation sites were predicted using NetNGlyc -1.0 (https://services.healthtech.dtu.dk/services/NetNGlyc-1.0/).

### 2.4 β-Mannanase Activity

β-mannanase activity was assessed using the *p*-hydroxybenzoic acid hydrazide (PAHBAH) method for reducing carbohydrates, with some modifications,^25^ briefly as follows. The PAHBAH reagent was prepared by combining 5% PAHBAH, 1 M bismuth nitrate, and 0.5 M NaOH. First, to evaluate the mannanolytic activity of each of the putative β-mannanases, the amount of reducing sugars released by each β-mannanase was assessed using 0.5% w/v ivory nut mannan as substrate in assay reactions of 30 min at 25 °C and pH 6.0 (50 mM sodium acetate buffer), and the enzymes were added at concentrations 0.15 mg/mL. All enzyme activity assays from Sections 2.5 to 2.8 were also conducted in 50 mM sodium acetate buffer at the chosen pH according to each enzyme, with 1% ivory nut mannan and 0.2 μM of the purified enzyme in thermostated reaction mixtures in Eppendorf tubes under agitation in thermomixers (Eppendorf, Hamburg, Germany) at the desired temperature. All the analyses were performed in triplicates. After the desired hydrolysis time (initial rate), 5% PAHBAH reagent was added to the reaction mixture, and the reaction mixture was centrifuged. The supernatants were then transferred to 96-well plates and incubated at 70 °C for 10 min. The plates were read at 410 nm by using a microplate reader. All enzyme activity assays from Sections 2.5 to 2.8 were detected by this method. One unit of β-mannanase activity was defined as the amount of enzyme required to produce 1 μmol of reducing end carbohydrate per minute.

### 2.5 Optimal Reaction Conditions Study Thermostability Assessment

A two-level face-centered central composite design (FCCD) was performed to identify the optimal pH and temperature for the hydrolysis of ivory nut mannan by *Eg*Man5A, *Ja*Man5A, and *Sl*Man5A, and to study the influence of pH and temperature on the hydrolysis rate. Based on preliminary experiments (data not shown), the levels were set as follows: pH 4.5–6.5 and 55–75 °C for *Eg*Man5A; pH 3.5–5.5 and 55–75 °C for *Ja*Man5A; and pH 4.5–6.5 and 65–85 °C for *Sl*Man5A. This resulted in a total of eight different reaction combinations (each performed in triplicate), plus a center point (performed in sixtuplicate). All hydrolysis assays were conducted as previously described, with adjustments made to the reaction mixture to achieve the desired pH and temperature. JMP statistical discovery version 18.1.0 program was used to design the experiments and to fit and analyze the data. The significance of the results was established using the ANOVA test at *p* ≤ 0.05. The predicted optimum values by the models were experimentally validated in triplicates.

To evaluate thermostability, the purified enzymes underwent one hour of incubation at their respective optimal temperature and pH, as well as temperatures 10 °C above and below the optimum. After the incubation period, the residual activity was determined under optimum reaction conditions for each enzyme, as described above. The results of enzyme activity at initial rates were used to calculate the thermodynamic parameters according to a previous study.^26^

### 2.6 Kinetics Parameters

To ascertain the Michaelis–Menten kinetics of the purified enzymes, 2, 1, 0.5, 0.2, and 0.1% concentrations of ivory nut mannan were employed. The reactions were performed under optimal pH and temperature conditions and measured at the initial rate. Data analysis was performed using OriginPro 2017 (https://www.originlab.com/origin), employing nonlinear regression to fit the data to the Michaelis–Menten equation.

### 2.7 Activity Assessment on Different β-mannan Substrates

To explore the specificity of the purified enzymes and draw comparisons with GH26 endo-1,4-β-mannanase (*C. japonicus*) and the commercial enzyme BGM “Amano” 10, different substrates, including ivory nut mannan, low DP β-mannan (1,4-β-D-Mannan), glucomannan (konjac; low viscosity), and locust bean gum from *Ceratonia siliqua* seeds were used at 1%. Hydrolysis assays were performed in triplicate, adjusting the conditions to the optimal pH and temperature for the purified enzymes. For the commercial enzymes, the assays were conducted at pH 4.5 and 50 °C. The results of each enzyme activity were assessed at an initial reaction rate.

### 2.8 Açaí Seeds Hydrolysis

Given the high content of polyphenolic compounds in açaí seeds,^27^ knife-milled açaí seed samples were subjected to hydroethanolic extraction using 80% ethanol at 25 °C under agitation, for 30 min, repeated three times. After extraction, the recovered açaí seeds samples were ball-milled for 1 min to standardize particle size and improve reproducibility. The hydrolysis was then conducted using 1% açaí seeds and 1 μM purified enzymes in 500 μL reactions under the optimal pH and temperature for the purified enzymes. The commercial enzyme BGM “Amano” 10 was used for comparison, and the assays were conducted at pH 4.5 and 50 °C. The results of each enzyme activity were assessed at the initial reaction rate.

### 2.9 Statistical Analysis

One-way ANOVA and Tukey HSD test were performed with R, version 4.4.1 (R Foundation for Statistical Computing, Vienna, Austria) for the determination of statistical significance. Statistical significance was established at *p* < 0.05.

### 2.10 Structure Prediction Analysis and Molecular Docking Analysis

Sequences were aligned using MAFFT version 7 (https://mafft.cbrc.jp/alignment/server/index.html), and the alignment was visualized and analyzed using Jalview version 2 and figures were generated using the Web server ENDscript 2 (https://espript.ibcp.fr/ESPript/cgi-bin/ESPript.cgi). AlphaFold2 models of expressed proteins were built via ColabFold,^28^ selecting the model with the lowest energy for each enzyme. *Eg*Man5A structure prediction had a predicted Local Distance Difference Test (pLDDT) of 97.4 and a predicted Template Modeling Score (pTM) of 0.951, *Ja*Man5A prediction had a pLDDT of 87.6 and a pTM of 0.903, and SlMan5A had a pLDDT of 84.1 and a pTM of 0.847, and all the scores indicate high confidence of the residue structure.^29^ The docking analysis was performed using AutoDock Vina Version 1.2^30^ with mannotetraose (compound CID: 53477669) as the ligand. The models were visualized and analyzed using PyMOL Molecular Graphics System, Version 1.2r3pre, Schrödinger, LLC. For each ligand-enzyme complex, the docking data were ranked according to binding energy from lowest to highest (lowest being best). Then, the best fit was selected by visual inspection in PyMOL (Schrödinger Inc., NY, U.S.A.) using the complex known for 1QNO^31^ as a guide. Amino acids were identified based on residues involved in among the top four ranked hydrogen-bond interactions and through structural superimposition with PDB entries 4QP0, 1QNO, and 3WH9.

## 3 Results and Discussion

### 3.1 CUPP Clustering, Sequence Analysis, and Heterologous Expression

To discover fungal endo-1,4-β-mannanases potentially specialized in the hydrolysis of linear mannans from açaí seeds, we examined data from a previous study on the proteome dynamics during different stages of açaí seed germination.^21^ This approach leverages the fact that plant enzymes required for hydrolyzing linear mannan storage during germination may provide good sequence inputs for identifying uncharacterized and putative fungal β-mannanases more specialized in linear mannan hydrolysis. As the açaí genome is currently unavailable, the proteomic analysis mentioned earlier^21^ identified the proteins using the phylogenetic family of the açaí (Arecaceae) database as a reference. Five uncharacterized or putative endo-β-mannanases homologues to açaí endo-β-mannanases were observed during germination (Figure S1). These five enzymes—XP_010911540.1 and XP_010943133.2 from *Elaeis guineensis* (oil palm), and XP_008805817.3, XP_008778491.2, and XP_017700190.1 from *Phoenix dactylifera* (date palm)—belong to the GH5 family, subfamily 7. XP_010911540.1 is homologous to XP_008805817.3, and XP_010943133.2 is homologous to XP_008778491.2. All were classified by CUPP as endo-1,4-β-mannanases (EC 3.2.1.78) and grouped in the same CUPP cluster: GH5:36:1. These enzymes served as the reference for fungal enzyme discovery in this study.

Initially, fungal candidate enzymes were identified through sequence alignment. The sequences with the highest similarities were further analyzed using CUPP, a nonalignment-based tool that is based on organizing the enzyme proteins into groups that share a similar motif group. The motif groups are based on peptide fragments of eight amino acids of which two are ambiguous;^18^ this clustering also enables functional annotation of the members of CAZyme families that contain enzymes of different functions (as in the case of the GH5 family of CAZymes).^19,22^

The aligned fungal sequences were also classified as endo-1,4-β-mannanases (EC 3.2.1.78) within the GH5 family, subfamily 7. While the majority was assigned to different CUPP groups, some were annotated as belonging to the same CUPP group as the plant β-mannanases, the GH5:36.1 (data not shown). Five fungal sequences (XP_007324538.1, KAF8922466.1, KAF5375179.1, KDQ56244.1, and KAF8642254.1) from the GH5:36.1 CUPP group were selected for synthesis, cloning, and expression (Figure S2). It is important to note that subsequent updates to the CUPP database, with the addition of over 1000 fungal genomes, have refined the CUPP clustering process.^22^ As a result, the fungal and plant sequences that were initially classified in the GH5:36.1 CUPP group have since been reassigned to different groups. However, their previous classification within GH5:36.1 still indicates a significant degree of peptide similarity among them.

From the plant and fungal sequences that were selected based on the previous analysis, only *Eg*Man5A (XP_010911540.1) from oil palm (plant), KDQ56244.1 (*Ja*Man5A) from *Jaapia argilacea* (fungus), and XP_007324538.1 (*Sl*Man5A), and from *Serpula lacrymans* (fungus), were successfully expressed recombinantly in *P. pastoris*. After expression, the enzymes were purified as depicted in Figure S3. The predicted molecular masses for *Eg*Man5A, *Ja*Man5A, and *Sl*Man5A were 41.5, 49.2, and 49.7 kDa, respectively, and gel analysis indicated that the purified *Eg*Man5A exhibited the expected mass, while the other two purified proteins demonstrated higher molecular masses than expected, likely due to glycosylation. In fact, N-glycosylation site prediction for *Ja*Man5A and *Sl*Man5A resulted in 5 sites, and none for *Eg*Man5A (data not shown). Consequently, both fungal proteins were subjected to enzymatic deglycosylation using EndoH and displayed the expected mass after treatment (Figure S3C). Since protein glycosylation is related to folding and stabilization, which directly influence the half-lives, degradation, and denaturation of enzymes,^32^ it is possible that the glycosylation of *Ja*Man5A and *Sl*Man5A could impact their overall stability. Additionally, the expression of the target enzymes was confirmed through Western blot analysis using anti-his tag monoclonal antibodies (Figure S3D). To confirm the activity of the expressed enzymes, endpoint extended assays were performed using ivory nut mannan as a substrate (data not shown). All three enzymes exhibited mannanolytic activity, releasing reducing sugars as products.

### 3.2 Optimization of the Reaction Conditions for *Eg*Man5A, *Ja*Man5A, and *Sl*Man5A, Thermostability and Kinetics

**Figure 1:**
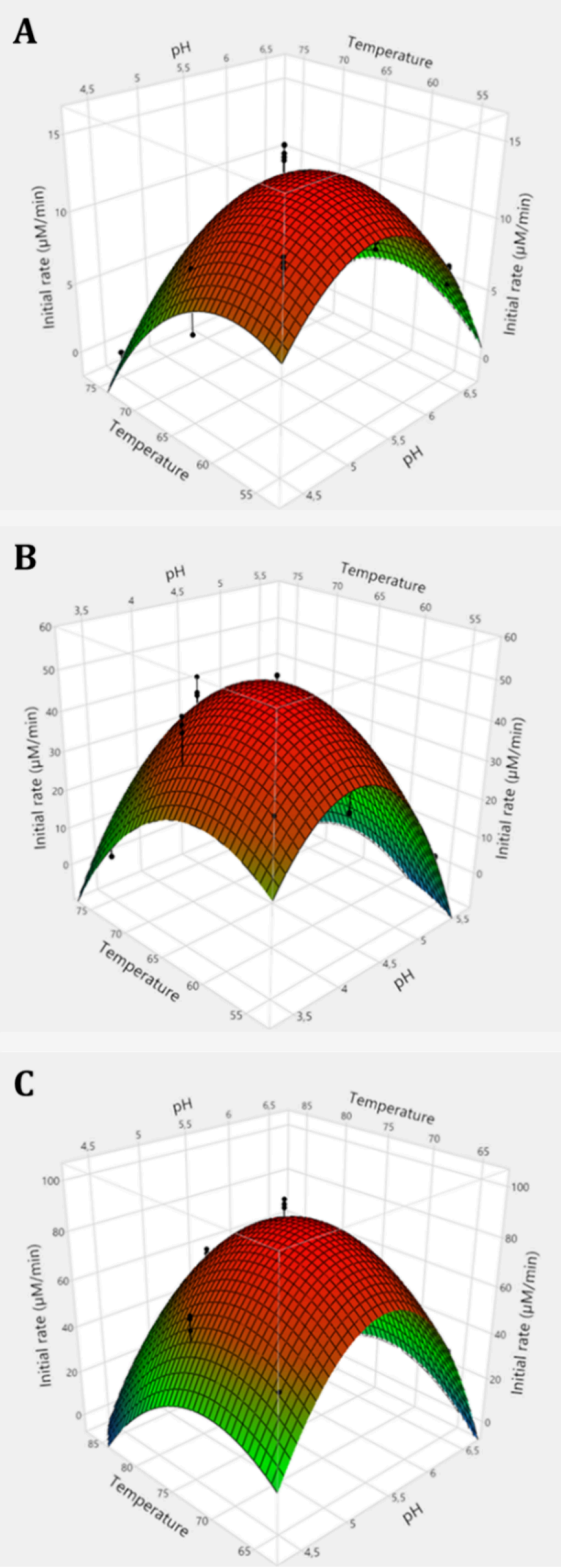
Response surface plots of initial rates (μM/min) of ivory nut (1%) hydrolysis by (A) *Eg*Man5A, (B) *Ja*Man5A, and (C) *Sl*Man5A as functions of pH and temperature. Face centered central composite design to predict optimal pH and temperature. The black dots represent the actual experimental data used to build the model. Resulted equations from modeling were (i) *Eg*Man5A: *Y*_1_ = 12.69 – 0.88*x*_1_ – 4.85*x*_1_^2^ – 3.98*x*_2_ – 3.85*x*_2_^2^ + 1.41*x*_1_*x*_2_; (ii) *Ja*Man5A: *Y*_1_ = 48.44 – 11.24*x*_1_ – 24.10*x*_1_^2^ – 4.31*x*_2_ – 13.34*x*_2_^2^ + 7.08*x*_1_*x*_2_; and (iii) *Sl*Man5A: *Y*_1_ = 87.02 – 5.03*x*_1_ – 41.12*x*_1_^2^ – 12.81*x*_2_ – 21.87*x*_2_^2^ + 6.70*x*_1_*x*_2_.

The impact of pH and temperature on enzyme activity was investigated using ivory nut mannan as substrate due to its chemical and structural similarity with açaí seeds mannan. The data were analyzed through an experimental design using an experimental design (an FCCD) model combining reaction conditions within a frame of pH and temperature ranges based on preliminary experiments for each enzyme. The results were depicted in a response surface plot (Figure 1).

According to the model, *Eg*Man5A has an optimum pH of 5.3 and temperature of 59 °C (Table 1), which is higher than the GH5_7 *Glycine max* (soybean) β-mannanase that has been reported to have optimum activity at 50 °C,^33^ but lower than the reported *Cocos nucifera* (coconut palm) β-mannanase optimum temperature of 70 °C.^34^*Ja*Man5A and *Sl*Man5A had optimal activity at 63 and 72 °C, and at pH 4.2 and 4.4, respectively (Table 1); these optima are comparable with those reported for other GH5 fungal β-mannanases.^35,36^ The expected initial rate when hydrolyzing ivory nut mannan under optimal conditions predicted by the models (Figure 1) was experimentally validated for *Eg*Man5A and *Sl*Man5A, corresponding to 13 and 92 μM/min (Table 1). Meanwhile, *Ja*Man5A, for which the model had a *R*^2^ of 0.88, exhibited an experimental initial rate of 55 μM/min, which was slightly higher than the predicted value (Table 1).

**Table 1:** Optimal Conditions for the Hydrolysis of Ivory Nut Mannan (1%)^a^

enzyme	optimum pH	optimum temperature (°C)	predicted initial rate (μM/min)	*R*^2^ and *p*-value	experimental initial rate (μM/min)	model error
*Eg*Man5A	5.3	59	13.9 ± 0.6	0.90 and <0.05	13.2 ± 0.2	5%
*Ja*Man5A	4.2	63	50.4 ± 2.5	0.88 and <0.05	55.7 ± 1.0	–10%
*Sl*Man5A	4.4	72	89.2 ± 2.3	0.95 and <0.05	91.6 ± 5.8	–3%

a The predicted initial rate obtained from a face-centered central composite design, the model’s statistical parameters, and the experimental initial rate achieved under optimal conditions.

The enzyme stability, at the optimum temperature and 10 °C below and above, was analyzed by assessing residual initial rates during 1 h incubation (Figures S4–6). The results were employed to estimate thermodynamic parameters, including the melting temperature (*T*_m_) and the half-life for each enzyme, as presented in Table 2.

**Table 2:** Half-lives and Melting Temperature (*T*_m_) of Enzymes at Optimum Temperature and ±10 °C

enzyme	temperature (°C)	half-life*t*_1/2_ (min)	*T*_m_ (°C)
*Eg*Man5A	**50**	139	60
	**60**	28	
	**70**	2	
*Ja*Man5A	**55**	315	70
	**65**	31	
	**75**	12	
*Sl*Man5A	**60**	990	78
	**70**	65	
	**80**	11	

*Eg*Man5A required around 2 h at 50 °C to reach half of the native initial rate. Comparatively, *Eg*Man5A demonstrated higher thermostability than β-mannanases from soybean, which lost 80% of activity when monitoring it for 0.5 h at 50 °C, and from *Arabidopsis thaliana* in 2 h lost 70% of activity at 45 °C.^33,37^ When increasing temperature, at 60 and 70 °C, to have comparable conditions to *Sl*Man5A, *Eg*Man5A became a less stable enzyme, presenting a half-life approximately 30 times lower than *Sl*Man5A. One of the factors that influence thermal stability is N-glycosylation,^38^ and the N-glycosylation prediction indicated no N-glycosylation site for *Eg*Man5A and 5 sites for *Sl*Man5A. *Ja*Man5A presented a half-life of 5 h at 55 °C, and *Sl*Man5A presented a half-life of 16.5 h at 60 °C. *Ja*Man5A showed a similar half-life of a GH5 β-mannanase from *Trichoderma asperellum*, which had a half-life of 4 h at 55 °C.^36^ Similar thermal stability to *Sl*Man5A was found in a β-mannanase from *Aspergillus niger* that maintained a high residual activity at 60 and 70 °C in 2 h of incubation.^39^ All three enzymes were thermostable during the assayed time at the lower temperature tested for each enzyme (Table 2). Regarding *T*_m_, *Eg*Man5A was the least thermostable enzyme, with a *T*_m_ of 60 °C. For *Ja*Man5A and *Sl*Man5A values, *T*_m_ were 70 and 78 °C, respectively. Similar *T*_m_ values have been reported for a β-mannanase from *A. niger* and its mutants.^40^ Among the tested β-mannanases, *Sl*Man5A showed the highest thermal stability.

**Figure 2:**
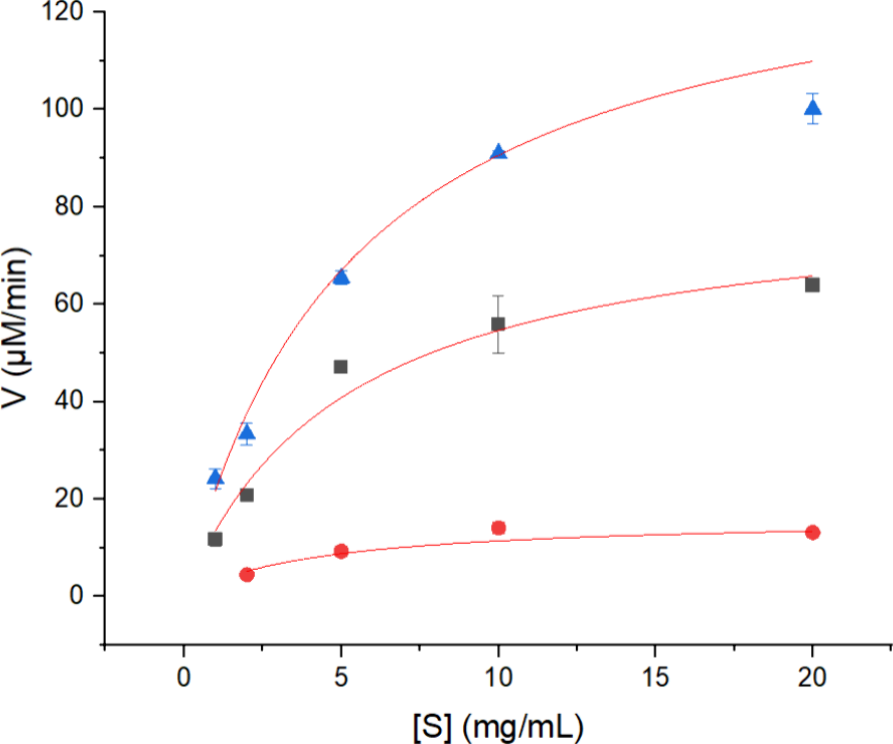
Influence of ivory nut concentration on initial rates (V and μM/min) at optimal temperatures and pHs. Blue triangles: *Sl*Man5A: 70 °C and pH 4.4; Black squares: *Ja*Man5A: 65 °C and pH 4.2; Red circles: *Eg*Man5A at: 60 °C and pH 5.3; Red lines represent Michaelis Menten modeling, with adjusted *R*^2^ of 99% (*Sl*Man5A), 95% (*Ja*Man5A), and 91% (*Eg*Man5A).

Subsequently, the enzyme rates at different concentrations of ivory nut mannan were assayed at optimum conditions, plotted, and modeled using the Michaelis–Menten equation (Figure 2).

As observed in Figure 2, all three catalytic sites from the enzymes became saturated with around 10–20 mg/mL of ivory nut mannan. Variations in enzyme rates were noted upon calculation of the kinetic parameters (Table 3). The apparent Michaelis–Menten constant (*K*_M_) toward ivory nut mannan of all three enzymes was similar, while the catalytic constant (*k*_cat_) and the specificity constant (*k*_cat_/*K*_M_) were statistically different (Table 3). *Eg*Man5A thus displayed the lower *k*_cat_ of the three enzymes, and in turn, this enzyme also had the lowest *k*_cat_/*K*_M_ (Table 3). The lower *k*_cat_/*K*_M_ of *Eg*Man5A was expected since it was tested at the lowest temperature, where the rate of reaction would be expected to be lower, all other things being equal. Besides, *Eg*Man5A is a plant-derived enzyme, which is evolutionarily designed to maintain plant metabolism and act inside the cells.^41^ Thus, these enzymes act differently from fungal hydrolases, which are normally secreted and evolved to work as effectively as possible as part of a portfolio of enzymes to ensure a sufficient carbon supply to the fungus. A similar *k*_cat_/*K*_M_ (0.72 mg s^–1^ mL^–1^) was observed for soybean β-mannanase when testing it with locust bean gum as a substrate.^33^ The *k*_cat_/*K*_M_ of *Sl*Man5A was nearly double that of *Ja*Man5A, making *Sl*Man5A the most efficient enzyme expressed in hydrolyzing ivory nut mannan, with a *k*_cat_/*K*_M_ of 2.2 mg s^–1^ mL^–1^. It is important to note that the higher efficiency of *Sl*Man5A compared to *Ja*Man5A could be attributed to the higher optimum temperature of *Sl*Man5A, reflecting the higher assay temperature. When reporting the kinetic parameters of β-mannanases, most studies do not use ivory nut mannan as the substrate, hindering the comparison of this study with previous findings. Nevertheless, studies of GH5 fungal β-mannanases kinetics toward locust bean and konjac gums reported similar values of *K*_M_ and *k*_cat_.^39,42^ Besides, a study has shown kinetic parameters for a fungal GH5 β-mannanase toward ivory nut mannan and other β-mannan substrates and concluded that the enzyme was more efficient when hydrolyzing glucomannan and galactomannan substrates.^43^

**Table 3:** Enzyme Kinetics Parameters When Hydrolyzing Ivory Nut Mannan, Obtained from Michaelis Menten Modeling

enzyme	*K*_M_ (mg mL^–1^)	*k*_cat_ (s^–1^)	*k*_cat_/*K*_M_ (mg s^–1^ mL^–1^)
*Eg*Man5A	4.5 ± 0.8^*b*^	1.5 ± 0.0^*d*^	0.3 ± 0.1^*d*^
*Ja*Man5A	4.6 ± 0.2^*b*^	6.7 ± 0.2^*c*^	1.5 ± 0.1^*c*^
*Sl*Man5A	4.7 ± 0.1*^b^*	10.6 ± 0.3^*b*^	2.2 ± 0.0^*b*^

^*a*^Assays were performed at optimal pHs and temperatures of enzymes. *Eg*Man5A: 60 °C and pH 5.3; *Ja*Man5A: 65 °C and pH 4.2; and *Sl*Man5A: 70 °C and pH 4.4. Letters ^*b–d*^ indicate significant difference (*p* < 0.05) between values of each enzyme parameter.

### 3.3 *Eg*Man5A, *Ja*Man5A, and *Sl*Man5A Specificities on β-mannan Substrates

The specific activity (U/mg of enzyme) of the expressed enzymes against various β-mannan substrates was tested and compared to two commercially available β-mannanases (Table 4), BGM “Amano” 10 and a GH26 endo-1,4-β-mannanase from *C. japonicus*. Regarding the linear mannan substrates, ivory nut mannan was hydrolyzed by all enzymes, as well as the 1,4-β-D-mannan, which catalysis is facilitated when compared to ivory nut mannan due to its lower degree of polymerization (∼15 DP). *Ja*Man5A and BGM “Amano” 10 performed equally in both substrates. In contrast, *Sl*Man5A exhibited the highest specific activity toward ivory nut mannan and 1,4-β-D-mannan, with activities of 362 ± 29 and 645 ± 25 U/mg of protein, respectively. These activities were approximately 30 and 40% higher, respectively, than those of GH26 endo-1,4-β-mannanase, which had the second highest activity. *Eg*Man5A was the least active enzyme for both substrates.

**Table 4:** Comparison of Substrate Specificity (U/mg of Protein) of the Endo-1,4-β-mannanases *Eg*Man5A, *Ja*Man5A, and *Sl*Man5A and Two Commercially Available β-mannanases

enzyme	U/mg of protein				
	ivory nut	1,4-β-D-mannan	locust bean gum	konjac gum	Açaí seeds
*Eg*Man5A	33.2 ± 3.7^*d,y*^	57.0 ± 4.7^*d,x*^	80.2 ± 9^*c,w*^	39.5 ± 3.1^*b,y*^	1.1 ± 0.1^*d,z*^
*Ja*Man5A	133 ± 11^*c,x*^	276 ± 11*^c,w^*	ND	ND	8.2 ± 0.9^*c,y*^
*Sl*Man5A	362 ± 29*^a,x^*	645 ± 25^*a,w*^	ND	ND	23.7 ± 1.5^*a,y*^
BGM “Amano” 10	164 ± 9^*c,x*^	278 ± 14^*c,w*^	261 ± 15^*b,w*^	76.0 ± 8.2^*b,y*^	12.5 ± 2.6^*b,z*^
GH26 Megazyme	286 ± 11*^b,y^*	464 ± 21^*b,x*^	510 ± 48^*a,x*^	1261 ± 99^*a,w*^	NA

Letters ^*a–d*^ indicate significant difference (*p* < 0.05) between values of substrate specificity (columns). Letters *^w–z^* indicate significant difference (*p* < 0.05) between values of enzyme specificity (rows). Letter *^e^ ND, not detected; NA, not assayed*.

The specific activity against ivory nut mannan is reported in some studies, e.g., Lima et al.^44^ who produced a β-mannanase from *Penicillium citrinum* that showed 62 U/mg of specific activity toward ivory nut mannan. Besides, another study reported a low specific activity (2.7 U/mg) of a fungal β-mannanase, hypothesizing that the low activity was due to the crystallinity of ivory nut mannan.^45^ Thus, the results of this study of enzyme activity against linear mannans are highly relevant for understanding the specificity of enzymes for these substrates, which are much less studied.

Among the expressed enzymes, only *Eg*Man5A showed specific activity against locust bean and konjac gums, while *Ja*Man5A and *Sl*Man5A exhibited no catalytic activity against these substrates during the assay period (Table 4). GH26 β-mannanase displayed the highest specific activity toward these gums, whereas *Eg*Man5A was the lowest. Similarly, a study of *A. thaliana* β-mannanase reported low specific activity toward all substrates tested,^37^ while fungal β-mannanases show a broad range of activities against locust bean and konjac gums in the literature. For example, an *Aspergillus nidulans* β-mannanase exhibited a specific activity of 184.8 U/mg against galactomannan, whereas a *Bispora* sp. β-mannanase showed an activity of 3373 U/mg.^46,47^

The specific activity for açaí seeds was significantly lower compared to the other substrates. This is likely because, unlike the other substrates, which were pure β-mannans, the entire aça seeds were processed by milling, incorporating not only the endosperm but also other components as the tegument. This mixture introduces a greater level of complexity compared to the other substrates (Table 4). Similarly, Pangsri et al.^48^ when testing the specific activity of a β-mannanase for different β-mannan substrates, found that the enzyme-specific activity toward defatted copra meal was 20 times lower than toward konjac gum. Despite the low absolute values, *Sl*Man5A displayed the highest specific activity against açaí seeds, indicating higher specificity to açaí seed linear mannan compared to the other enzymes, which could also be associated with its higher thermal robustness. This is consistent with its higher specificity toward ivory nut mannan and 1,4-β-D-mannan. To the best of our knowledge, no study has reported the specific activity of enzymes toward untreated açaí seeds. However, a recent study reported a 10% yield of enzymatic hydrolysis after 24 h using the enzyme RohalaseGMP (AB Enzymes).^17^ Another study also reported a low yield (less than 3%) when hydrolyzing untreated açaí seeds after 72 h using BGM “Amano” 10.^3^

Thus, our strategy of using one of the endo-β-mannanases identified in a previous study on proteomic dynamics during açaí germination^21^ as a reference was successful in capturing fungal enzymes with high specificity to linear mannans. However, the plant endo-β-mannanase exhibited a broader action toward different β-mannan substrates (Table 4). Given that these differences could be attributed to subtle variations in the catalytic sites of the enzymes, we proceeded with the investigation of structural models of the catalytic domains.

### 3.4 *Eg*Man5A, *Ja*Man5A, and *Sl*Man5A Structural Modeling

Structural AlphaFold2 models of *Eg*Man5A, *Ja*Man5A, and *Sl*Man5A were generated. The amino acid residues present in the active site and subsite positions of *Eg*Man5A were predicted by superimposition with the PDB structure of *Solanum lycopersicum* endo-1,4-β-mannanase (1RH9), while those for *Ja*Man5A and *Sl*Man5A were predicted by using the PDB structure of *Rhizomucor miehei* (4QP0). Additionally, the protein sequence alignment between the three expressed endo-1,4-β-mannanases and all GH5 endo-1,4-β-mannanases sequences with structures available at PBD database was analyzed (Figure S7).

**Figure 3:**
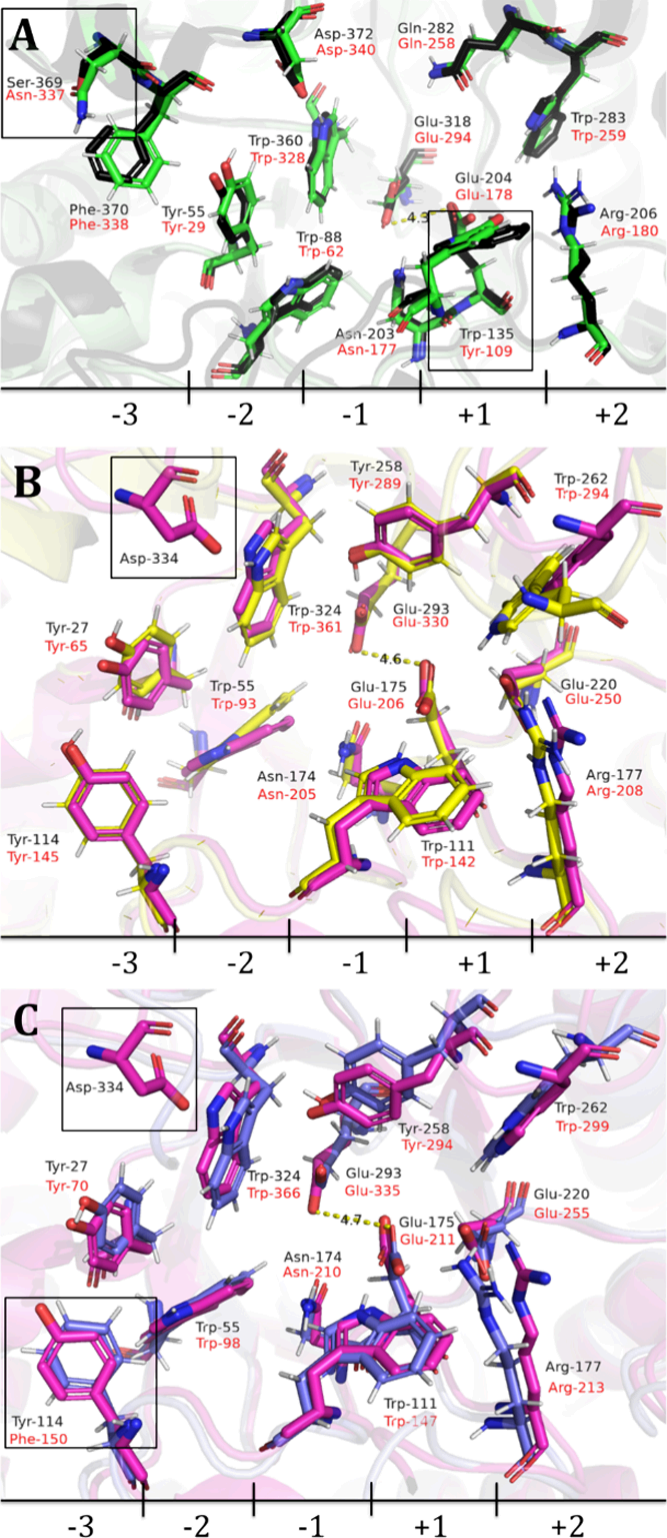
Amino acid residues present in the active site of (A) *Eg*Man5A, (B) *Ja*Man5A, and (C) *Sl*Man5A. The active site and the subsite positions of *Eg*Man5A (green) were predicted (amino acids names in red) by superimposition with the PDB structure of *Solanum lycopersicum* endo-1,4-β-mannanase (1RH9) (black) (amino acids name in black), while those for *Ja*Man5A (yellow) and *Sl*Man5A (purple) (amino acids names in red) were predicted using the PDB structure of *Rhizomucor miehei* endo-1,4-β-mannanase (4QP0) (pink) (amino acids names in black). Black boxes indicate the differences found in the superposition of the active sites.

The active site of *Eg*Man5A matched closely with the model from *S. lycopersicum* (1RH9) when analyzing it by superimposition (Figure 3). The amino acid residues present in the catalytic active site of 1RH9 were predicted by an earlier study, that used mannopentaose as substrate.^49^*Eg*Man5A exhibited almost all the residues present in 1the RH9 site, except for two residues, a Trp-135 located in the +1 subsite, that in *Eg*Man5A was substituted for a Tyr-109, and a Ser-369 located in the −3 subsite that was substituted by an Asn-337 (Figure 3A).

The substitution of serine with asparagine should not alter the reactions of the catalytic cleft, as both amino acids are polar and uncharged. Additionally, analysis of the alignment shows that this region (position 343) is not conserved among the aligned β-mannanase sequences (Figure S7). In contrast, the change in tryptophan to tyrosine involves more significant differences. While both are aromatic amino acids, tyrosine’s hydroxyl group confers higher polarity, whereas tryptophan is more hydrophobic and bulkier due to the indole ring in its side chain. Moreover, Trp-135 is a conserved amino acid among GH5_7 β-mannanases, located at position 109 in the alignment (Figure S7). This change could impact the overall efficiency or specificity of this enzyme. Indeed, *Eg*Man5A was the least efficient enzyme when comparing the results of kinetics and enzyme activity (Tables 3 and 4). However, changes from Trp to Tyr do not always affect the catalytic performance, and the impact of this change should be evaluated experimentally. For example, a study on a mutant form of α-1,3 galactosyltransferase with conserved Trp to Tyr substitution found no differences in overall structure and hydrolase activity, but it did impact the transferase efficiency.^50^ Additionally, the broader substrate specificity of *Eg*Man5A could be associated with the requirement for degradation and/or modification of different types of β-mannan that are stored during different phases of seed development and germination, as reported in the development of coffee beans.^51^

Most of the active site residues from *Ja*Man5A and *Sl*Man5A were completely congruent in spatial position when superimposed with 4QP0,^52^ except for Tyr-114 in *Sl*Man5A, which was substituted with phenylalanine, and the absence of Asp-334 from 4QP0 in both *Ja*Man5A and *Sl*Man5A (Figure 3B,C). Indeed, in the alignment, position 346 corresponds to an aspartic acid residue in the GH5_7 β-mannanases with available PDB structures, while *Ja*Man5A and *Sl*Ma5A have a serine in this position (Figure S7). Additionally, when comparing the superimposition of Asp-334 with fungal GH5_7 β-mannanase structures, the conserved position of this amino acid was evident. In contrast, the serine residues of *Ja*Man5A (Ser-386) and *Sl*Man5A (Ser-381) are in a different position and may not even be in the catalytic cleft (Figure S8).

Interestingly, *Ja*Man5A and *Sl*Man5A did not exhibit hydrolytic activity toward locust bean and konjac gum (Table 4). The lack of activity of these enzymes toward galactomannan and glucomannan was further discussed. The role of the aspartic acid residue in this position was briefly discussed in a previous study in which the structure of a GH5 β-mannanase was superimposed on a GH5 endoglucanase structure. It was suggested that this residue could be involved in differences observed in mannotriose and cellotriose selectivity.^53^ The aspartic acid residue in the −2 subsite was also found in the active site of *Eg*Man5A (Asp-372), which was also capable of hydrolyzing locust bean and konjac gums (Figure 3A; Table 4). Additionally, the six structures from GH5_7 fungal β-mannanases (4QP0, 4AWE, 3ZIZ, 3WFL, 1QNO, and 3WH9) all have a conserved aspartic acid at this position (Figure S8), and all of them were reported as able to hydrolyze galactomannans and/or glucomannans.^42,53,43,45,54,31,55^ Furthermore, a study that investigated the specificity of GH26 β-mannanases, and its correlation with mutations in catalytic subsites, found that subsites −2 and −1 are primarily responsible for galactomannan and glucomannan specificity in these enzymes.^15^ Therefore, the differences observed in the −2 region in *Ja*Man5A and *Sl*Man5A could be related to their lack of hydrolysis activity in locust bean and konjac gums.

**Figure 4:**
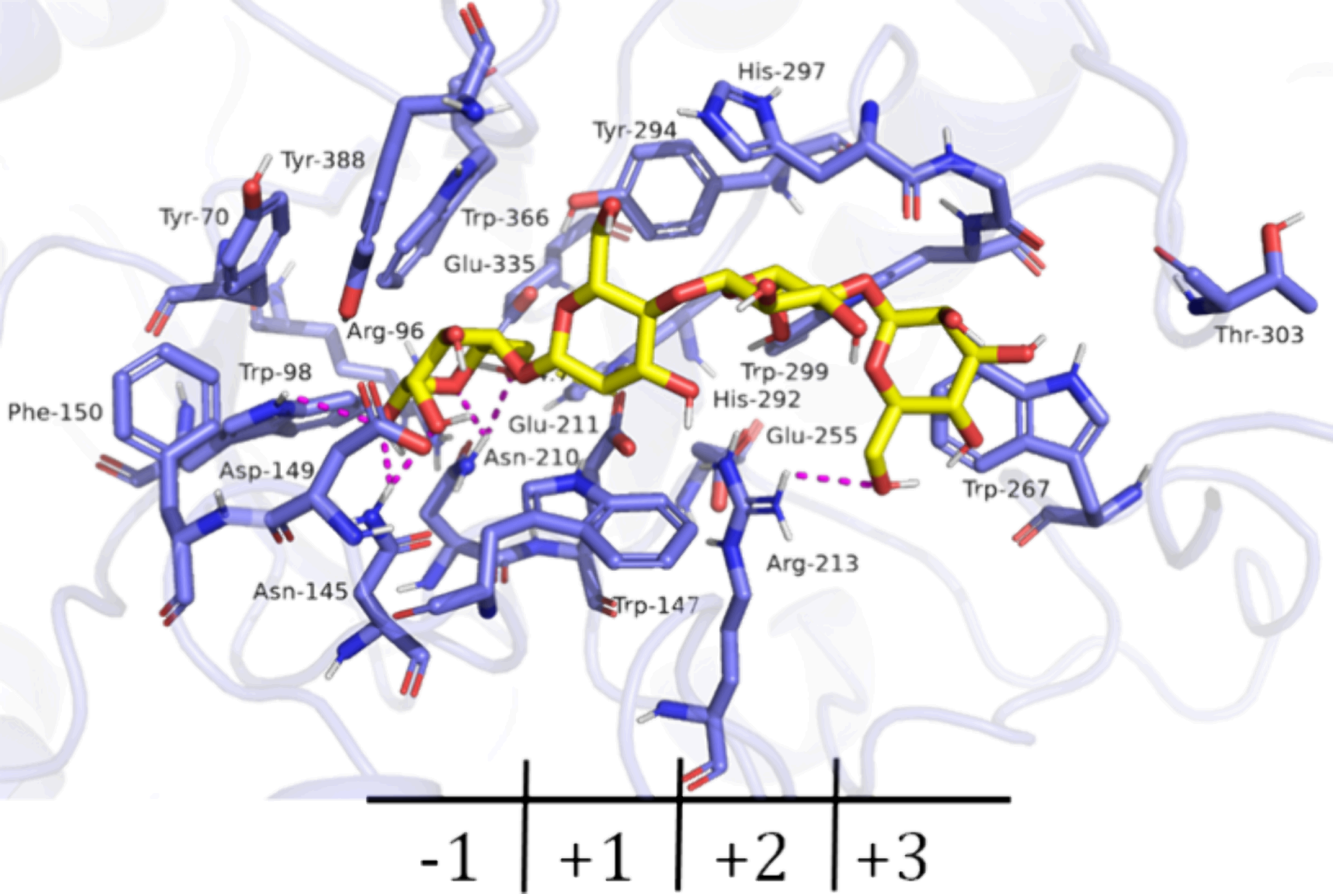
Active site of *Sl*Man5A. Interaction predicted by docking with mannotetraose (yellow sticks). Hydrogen-bond interactions are presented as magenta dotted lines.

*Sl*Man5A displayed high specificity in hydrolyzing 1,4-β-D-mannan, ivory nut β-mannan, and açaí seeds, likely due to its higher optimal temperature. Molecular docking revealed distinct interactions within its active site, including Asn-145, Tyr-388, His-297, and Thr-303 (Figure 4). While its superior thermal robustness may explain the enhanced hydrolysis performance, the observed variations in the active site suggest potential amino acids involved in linear β-mannan specificity. Further investigation is needed to confirm these findings.

In conclusion, *Eg*Man5A, *Ja*Man5A, and *Sl*Man5A were proven to be thermostable GH5 endo-1,4-β-mannanases that were active on linear mannan. *Sl*Man5A and *Ja*Man5A presented high specificity toward unsubstituted mannans, with *Sl*Man5A being the most efficient β-mannanase toward 1,4-β-D-mannan, açaí seeds, and ivory nut mannan with the highest *k*_cat_/*K*_M_ specific activities. This could have been influenced by the higher thermotolerance of *Sl*Man5A, which enabled catalysis at higher optimum temperatures. The difference in −2 and −1 subsites suggests a relationship with the lack of activity toward galactomannan and glucomannan hydrolysis by *Ja*Man5A and *Sl*Man5A, requiring further investigation. The high specificity and efficiency of *Sl*Man5A, combined with its enhanced stability at elevated temperatures, make it a promising candidate for biotechnological applications. Future studies will focus on validating these structural findings through targeted mutagenesis and exploring the broader substrate range of these enzymes in industrial processes.

The characterization of enzymes that are specific for the hydrolysis of ivory nut mannan and açaí seeds provides a basis for an improved understanding of the hydrolysis of linear mannan. This study hereby gives new insight that could pave the way for the future sustainable valorization of açaí seeds, turning waste into value and livelihood.

## Supporting Information Available

Figure S1: Alignment of Arecaceae family endo-1,4-β-mannanases sequences; Figure S2: Alignment of fungal endo-1,4-β-mannanases sequences; Figure S3: SDS-PAGE and western-blot analysis; Figure S4: Thermostability analysis of *Eg*Man5A; Figure S5: Thermostability analysis of *Ja*Man5A; Figure S6: Thermostability analysis of *Sl*Man5A; Figure S7: Alignment of GH5 endo-1,4**-**β-mannanases sequences; and Figure S8: Structural difference of negative subsites (PDF)
