# Supplementary material for: Discovery and Characterization of Mannan-Specialized GH5 Endo-1,4-β-mannanases: a Strategy for Açaí (Euterpe oleracea Mart.) Seeds Upgrading
Source: J Agric Food Chem. 2024 Dec 16;73(1):625–34. doi: 10.1021/acs.jafc.4c07018 (PMC11726631; doi:10.1021/acs.jafc.4c07018)
Supplement: Supplementary file 1 — jf4c07018_si_001.pdf [file jf4c07018_si_001.pdf]

Supporting information for

Discovery and characterization of mannan specialized GH5 endo-1,4-β-mannanases: a strategy for açaí (*Euterpe oleracea* Mart.) seeds upgrading

Roberta P. Espinheira[1,2], Kristian Barrett[3], Lene Lange[4], Ayla Sant'Ana da Silva[1,2]*& Anne S. Meyer[3]*

[1]Divisão de Catálise, Biocatálise e Processos Químicos, Instituto Nacional de Tecnologia, Av. Venezuela 82, 20081-312, Rio de Janeiro, Brazil.

[2]Programa de Pós-graduação em Bioquímica, Universidade Federal do Rio de Janeiro, Av. Athos da Silveira Ramos 149, 21941-909, Rio de Janeiro, Brazil.

[3]Department of Biotechnology and Biomedicine, Technical University of Denmark, Søltofts Plads 221, 2800, Kgs Lyngby, Denmark.

[4]LL BioEconomy, Research & Advisory, Karensgade 5, 2500, Copenhagen, Denmark.

* Corresponding authors' email address: ayla.santana@int.gov.br

asme@dtu.dk

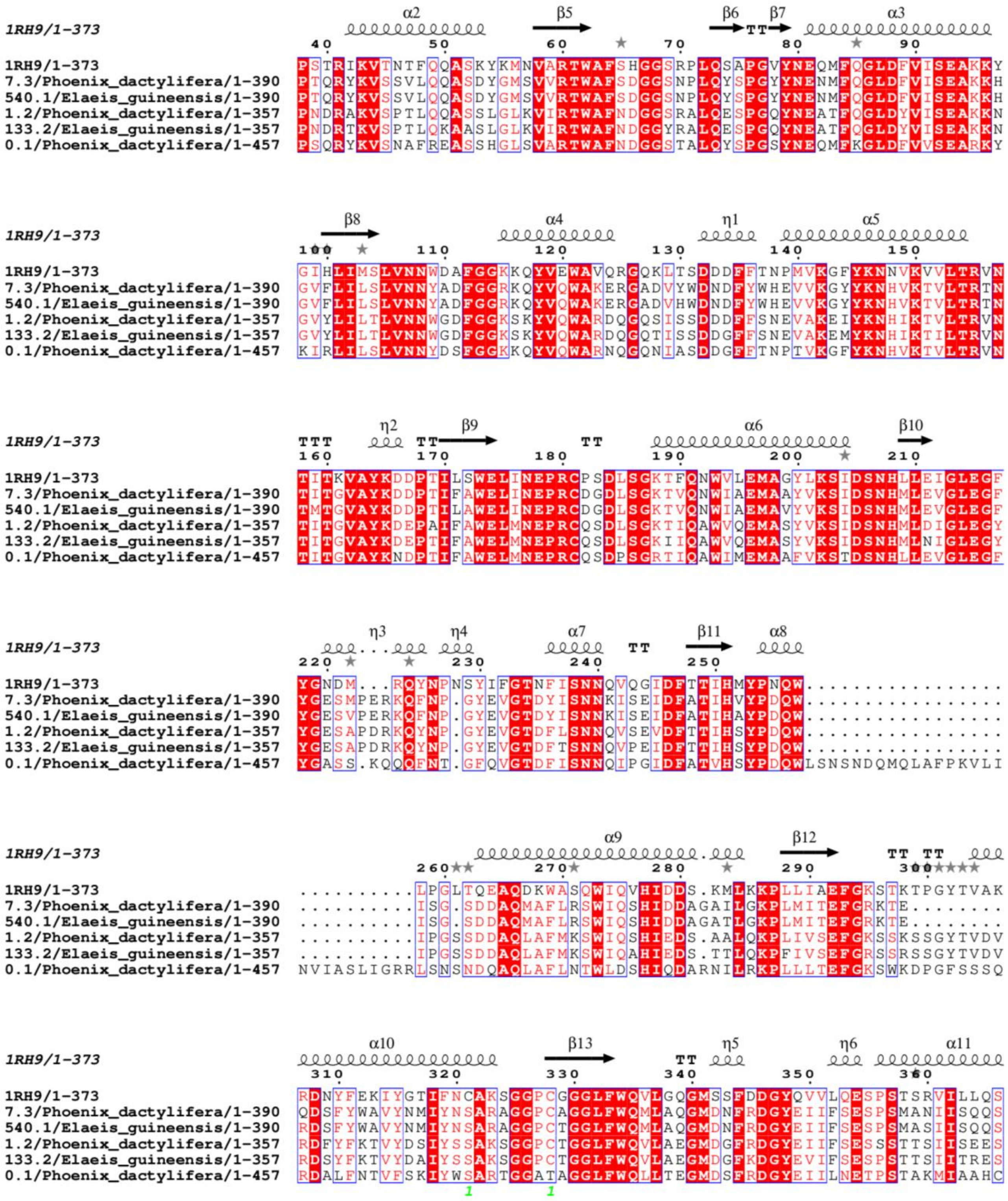

Figure S1. Alignment of Arecaceae family endo-1,4-β-mannanases sequences used in this study. The PDB structure 1RH9 was used for secondary structure depiction.

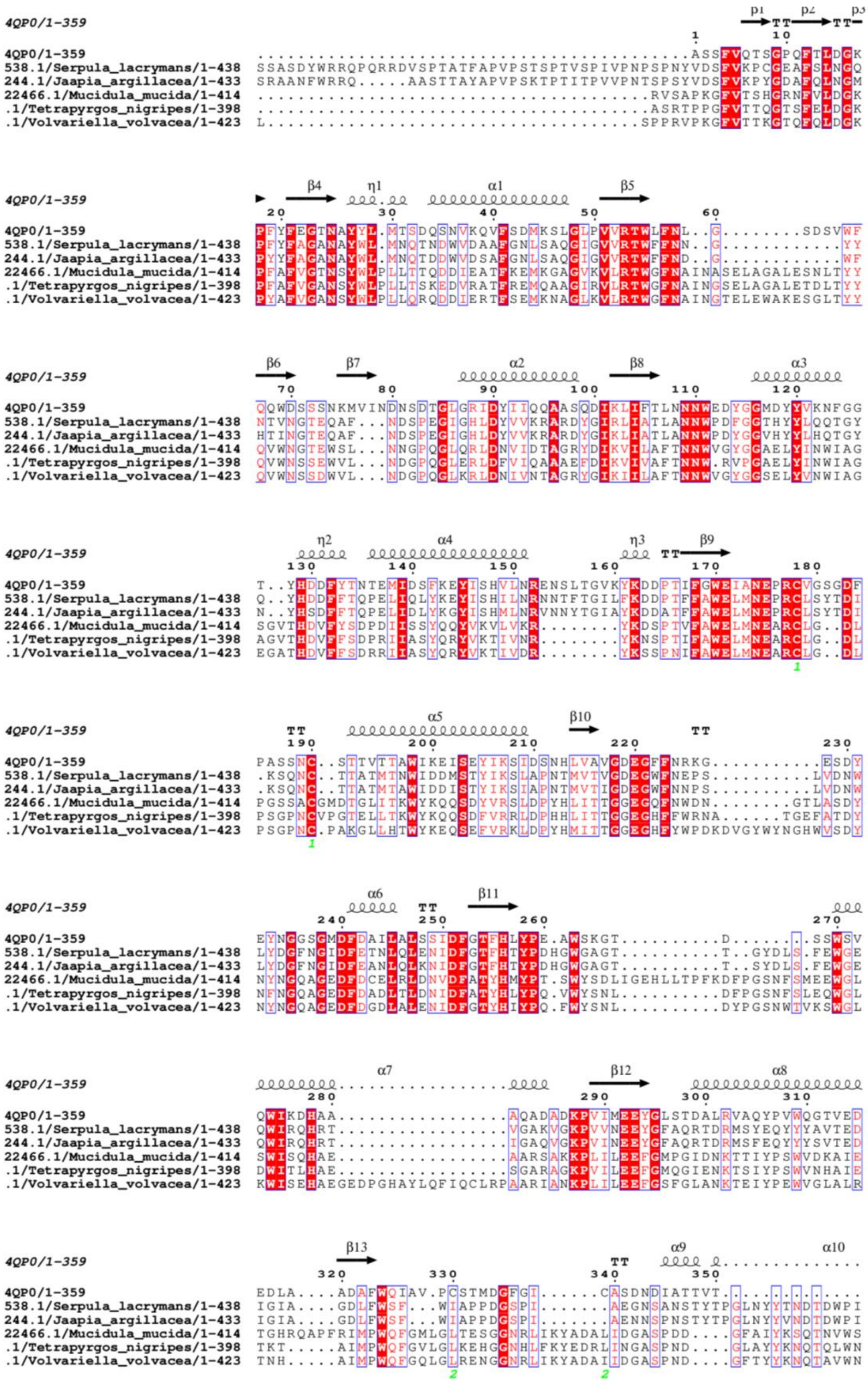

Figure S2. Alignment of fungal endo-1,4-β-mannanases sequences used in this study. The PDB structure 4QP0 was used for secondary structure depiction.

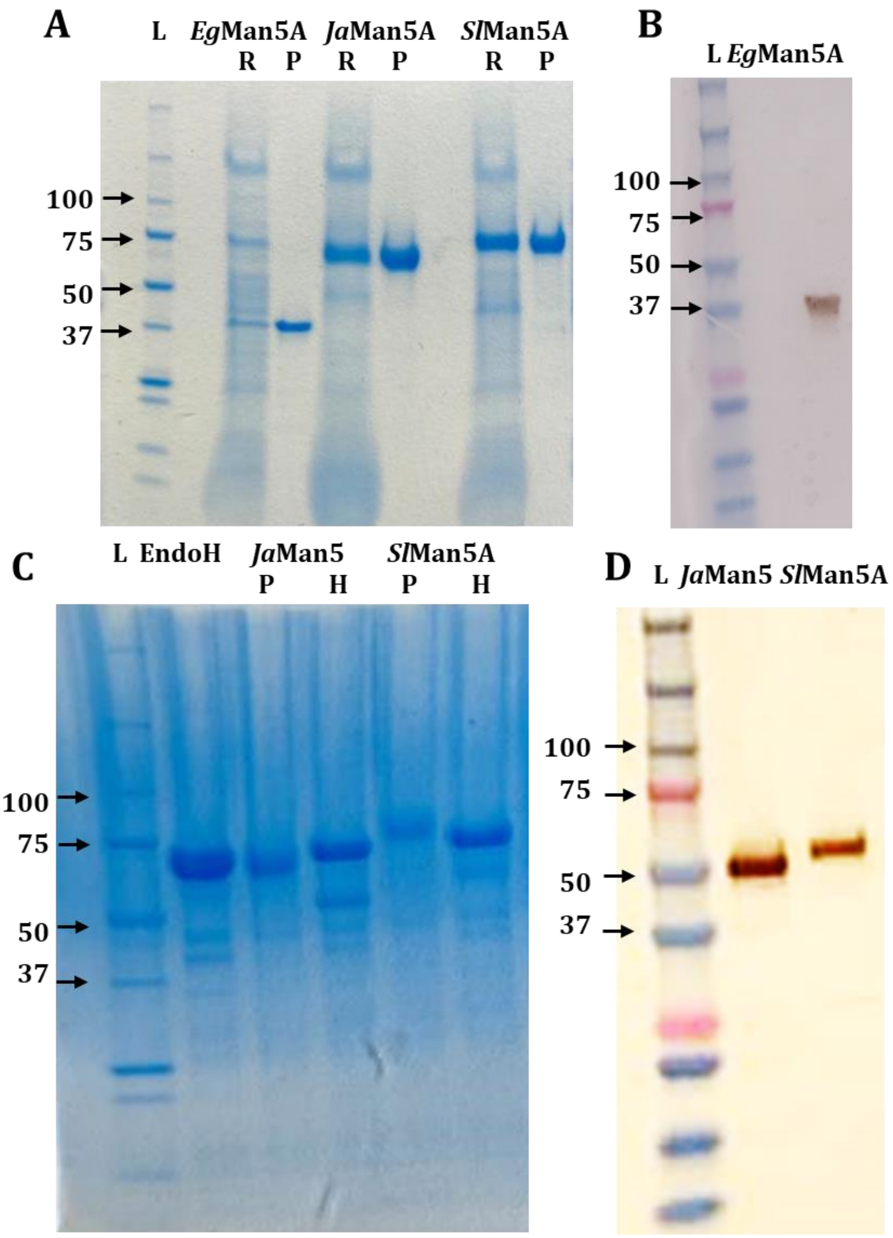

Figure S3. (A) SDS-PAGE of raw (R) and purified (P) fraction of *Eg*Man5A, *Ja*Man5A, and *Sl*Man5A. (B) Western-blot analysis of *Eg*Man5A after purification. (C) SDS-PAGE of *Ja*Man5A and *Sl*Man5A purified (P) and after EndoH hydrolysis (H). (D) Western-blot analysis of *Ja*Man5A and *Sl*Man5A after EndoH treatment.

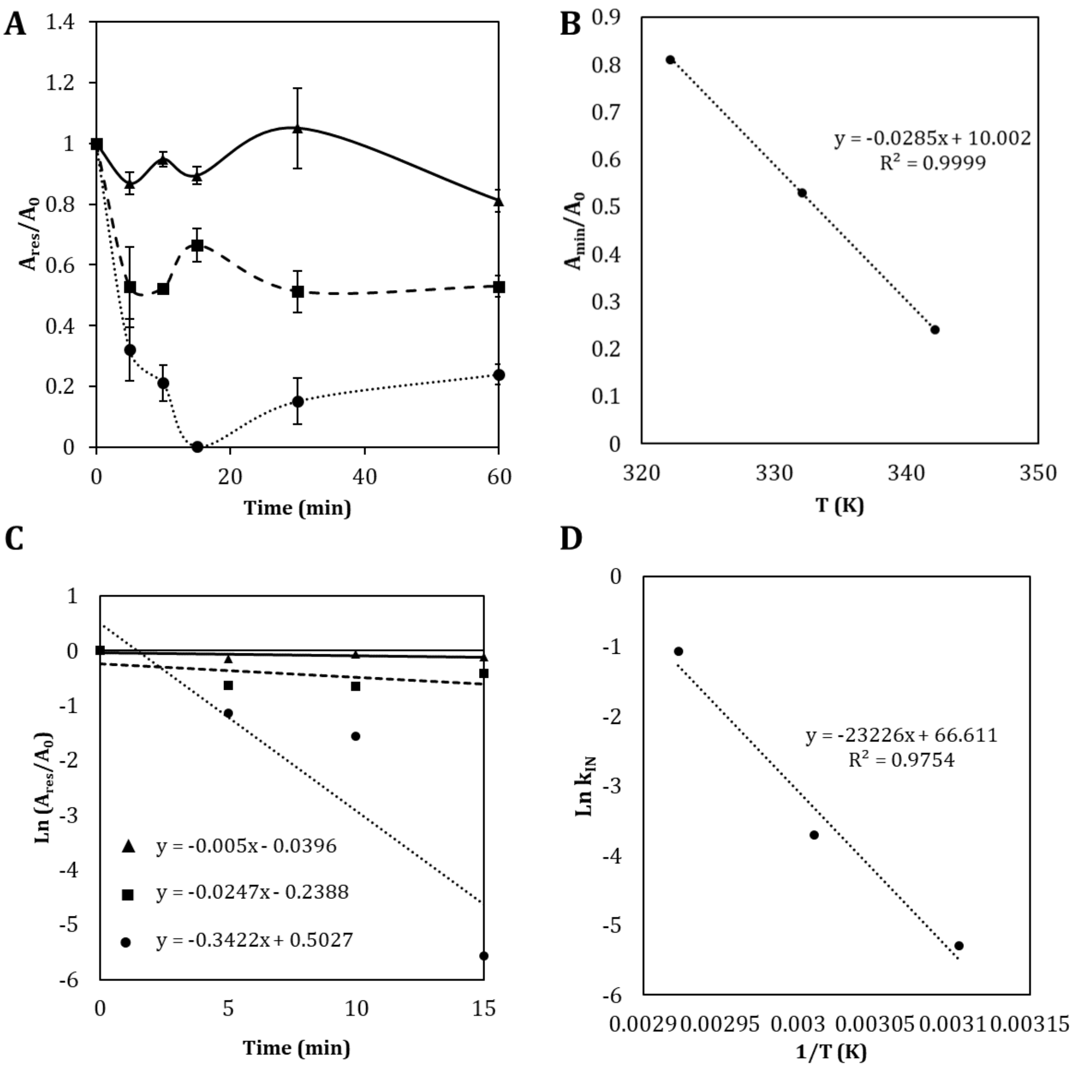

Figure S4. Thermostability analysis of *Eg*Man5A. (A) Ratio between residual activity and native activity obtained after incubation at 50 °C (▲), 60 °C (■), and 70 °C (●) during 60 min. (B) Melting temperature (Tm) estimation using the ratio of 60 min (A_{min}/A_0) and the three temperatures tested. Tm was considered as the temperature when the residual activity dropped down to 50% when compared to native activity. (C) Rates of thermal inactivation (k_{IN}) at 50 °C (▲), 60 °C (■), and 70 °C (●) during 15 min, obtained from the first order plot of figure A. The slopes represent the k_{IN}. (D) Arrhenius plot of ln k_{IN} vs. 1/T to obtain the activation energy of denaturation $E^{\#}_a$. Slope = - $E^{\#}_a$/R.

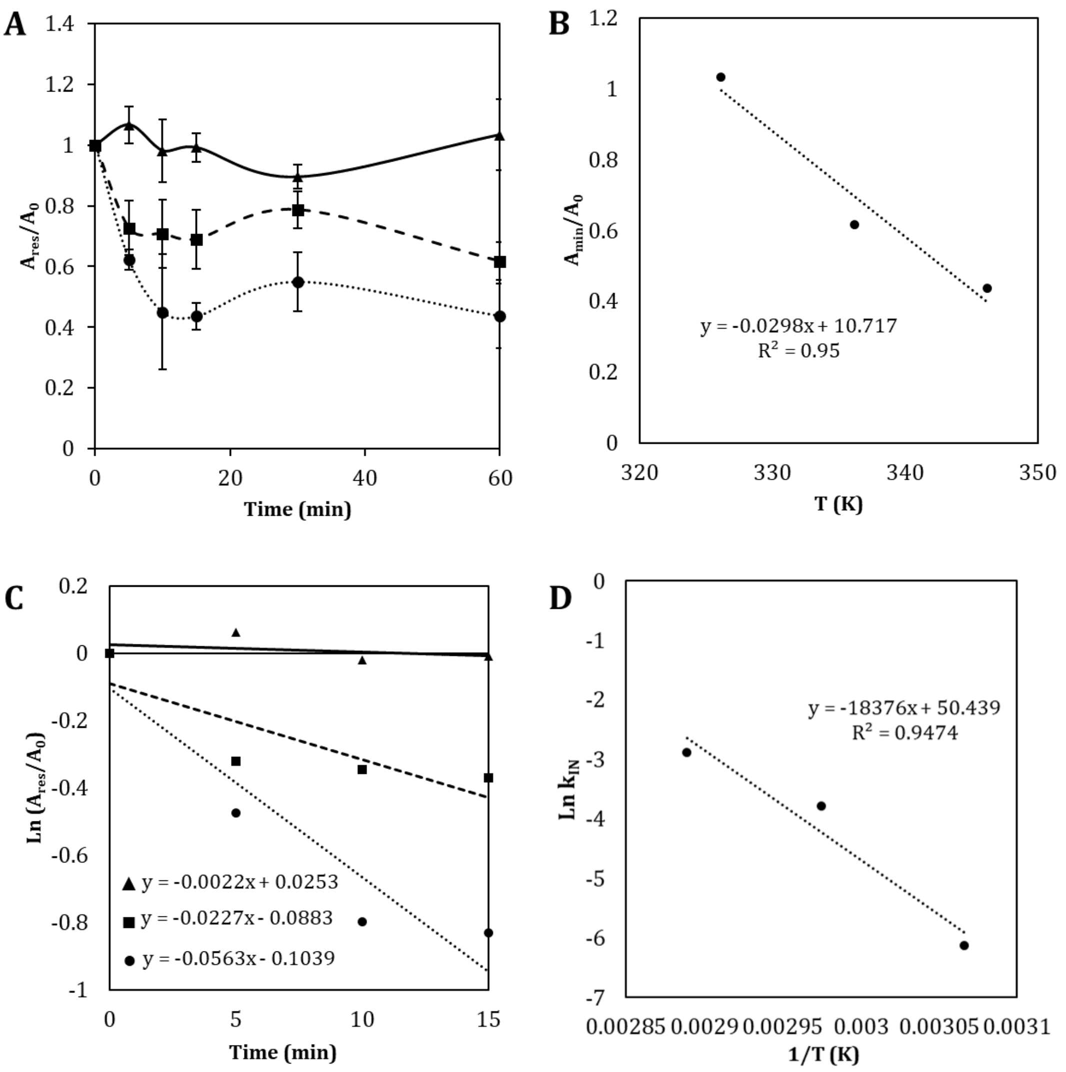

Figure S5. Thermostability analysis of *Ja*Man5A. (A) Ratio between residual activity and native activity obtained after incubation at 55 °C (▲), 65 °C (■), and 75 °C (●) during 60 min. (B) Melting temperature (Tm) estimation using the ratio of 60 min (A_{min}/A_0) and the three temperatures tested. Tm was considered as the temperature when the residual activity dropped down to 50% when compared to native activity. (C) Rates of thermal inactivation (k_{IN}) at 55 °C (▲), 65 °C (■), and 75 °C (●) during 15 min, obtained from the first order plot of figure A. The slopes represent the k_{IN}. (D) Arrhenius plot of ln k_{IN} vs. 1/T to obtain the activation energy of denaturation $E^{\#}_a$. Slope = - $E^{\#}_a$/R.

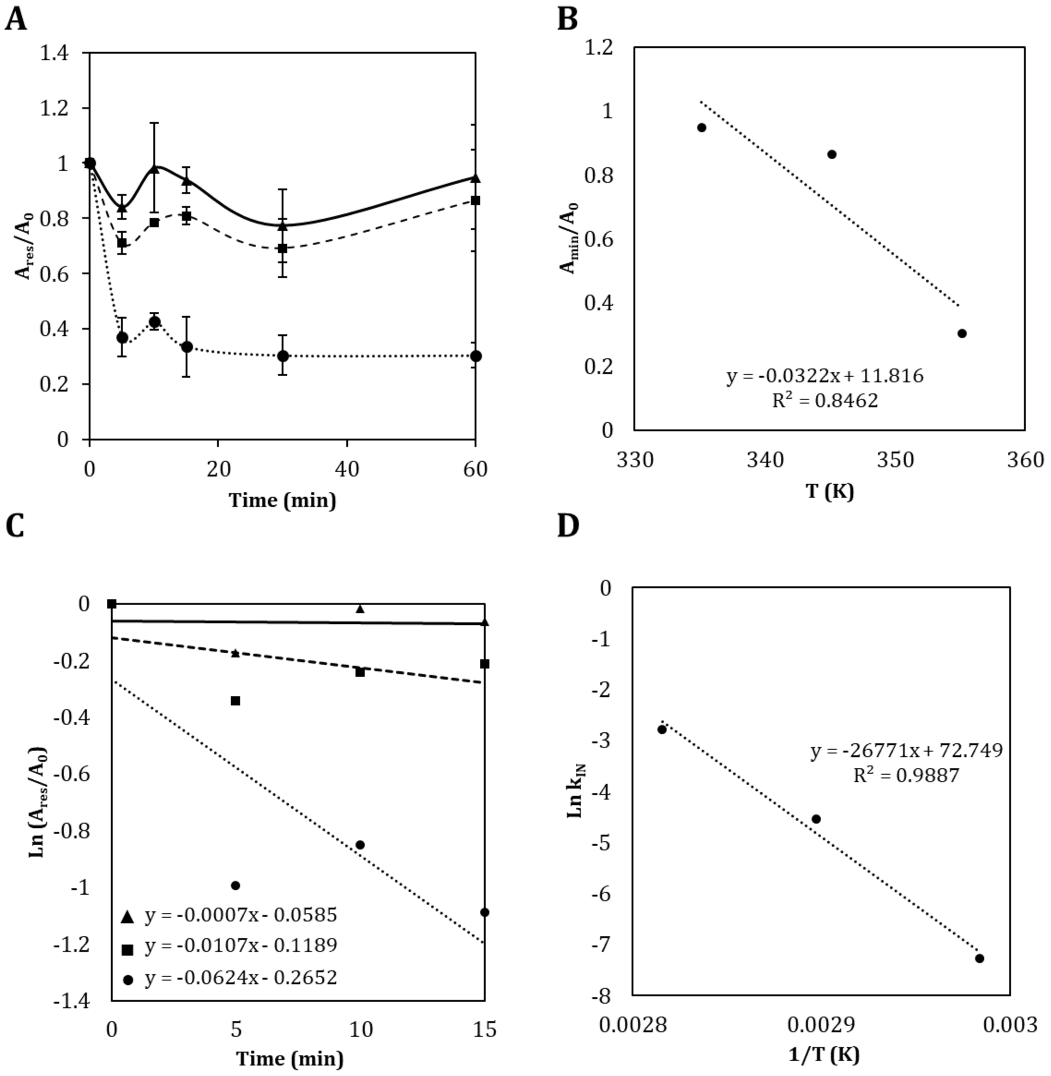

Figure S6. Thermostability analysis of *Sl*Man5A. (A) Ratio between residual activity and native activity obtained after incubation at 60 °C (▲), 70 °C (■), and 80 °C (●) during 60 min. (B) Melting temperature (Tm) estimation using the ratio of 60 min (A_{min}/A_0) and the three temperatures tested. Tm was considered as the temperature when the residual activity dropped down to 50% when compared to native activity. (C) Rates of thermal inactivation (k_{IN}) at 60 °C (▲), 70 °C (■), and 80 °C (●) during 15 min, obtained from the first order plot of figure A. The slopes represent the k_{IN}. (D) Arrhenius plot of ln k_{IN} vs. 1/T to obtain the activation energy of denaturation $E^{\#}_a$. Slope = - $E^{\#}_a$/R.

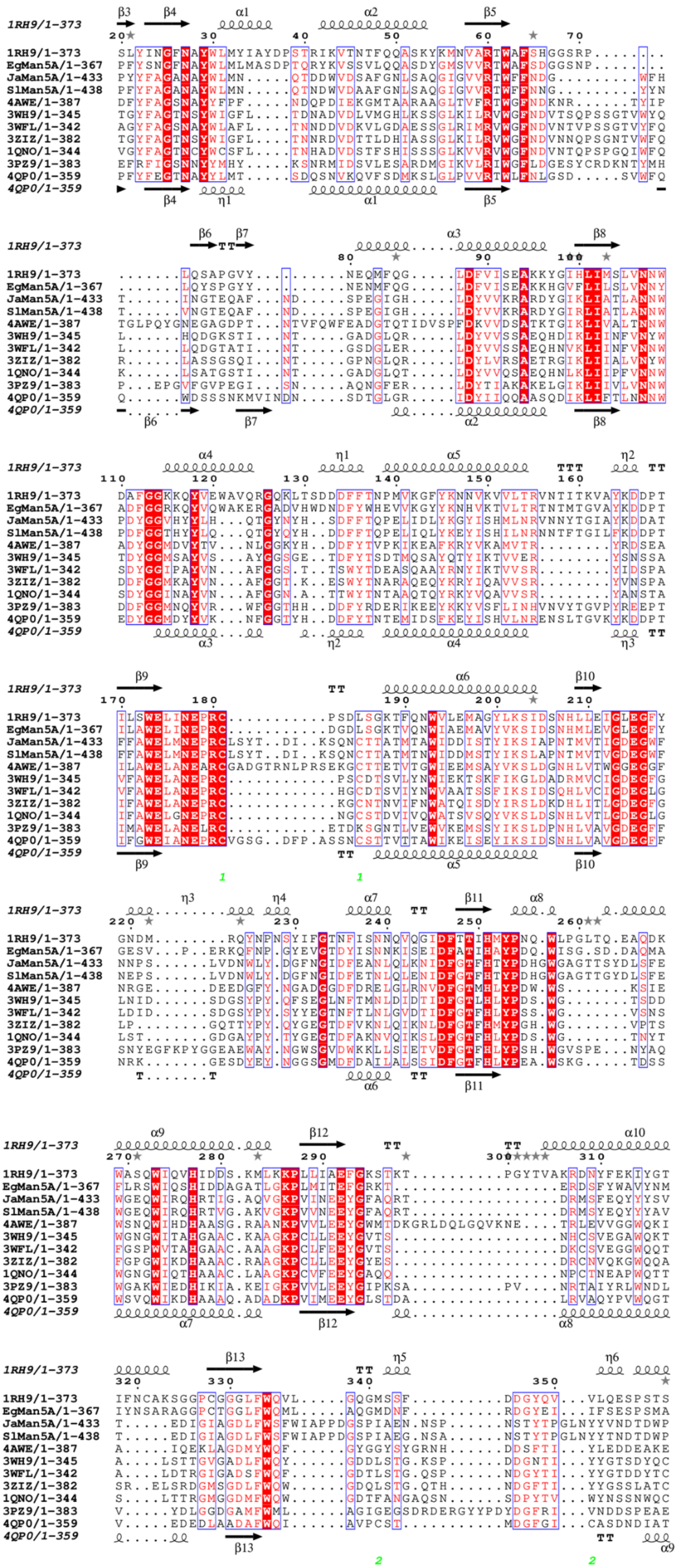

Figure S7. Alignment of GH5 endo-1,4-β-mannananases sequences with predicted structures available at PBD database. 1RH9: from *Solanum lycopersicum,* 4QP0: *Rhizomucor miehei*, 4AWE: *Neurospora sitophila,* 3ZIZ: *Podospora anserina,* 3WFL: *Talaromyces trachyspermus,* 1QNO: *Trichoderma reesei*, 3WH9: *Aspergillus niger* BK01, and 3PZ9: *Thermotoga petrophila* RKU-1.

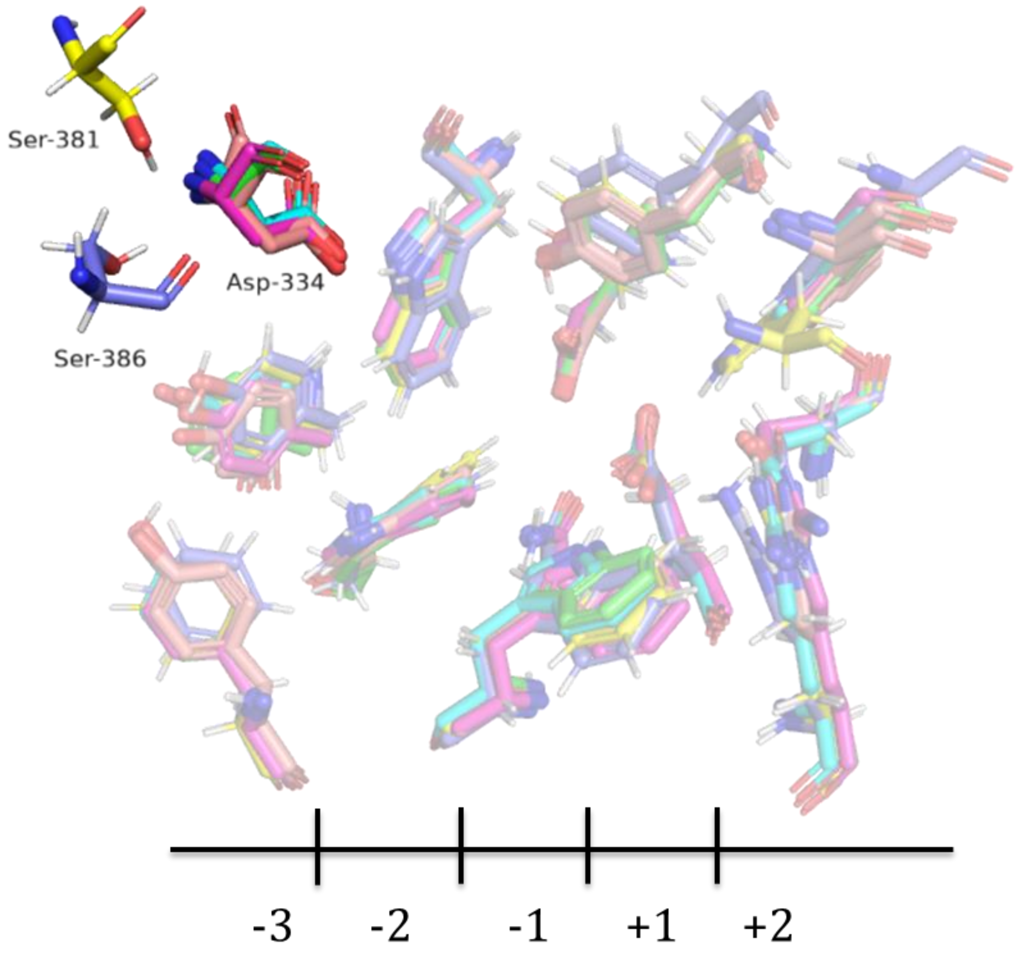

Figure S8. The structural difference of negative subsites between *Ja*Man5A, *Sl*Man5A, and GH5_7 endo-1,4-β-mannanases PDB available structures. The difference of aspartic acid (Asp-334) to serine residues at -3 and -2 subsites of *Ja*Man5A (yellow stick Ser-381) and *Sl*Man5A (blue stick Ser-386). Superimposition of fungal GH5 mannanases. Including *Ja*Man5A, *Sl*Man5A, 4QP0: *Rhizomucor miehei*, 4AWE: *Neurospora sitophila*, 3ZIZ: *Podospora anserina*, 3WFL: *Talaromyces trachyspermus*, 1QNO: *Trichoderma reesei*, 3WH9: *Aspergillus niger* BK01, and 3PZ9: *Thermotoga petrophila* RKU-1.
